# Inner Ear Schwannoma Treated With Intratympanic Gentamicin for Recurrent Ménière's Disease-Like Vertigo: A Case Report

**DOI:** 10.7759/cureus.116464

**Published:** 2026-09-18

**Authors:** Honoka Suzuki, Keiji Honda, Yoshimaru Mizoguchi, Natsuki Aoki, Ayako Nishio, Takamori Takeda, Takeshi Tsutsumi

**Affiliations:** 1 Department of Otolaryngology, Institute of Science Tokyo, Tokyo, JPN

**Keywords:** endolymphatic hydrops, inner ear schwannoma, intralabyrinthine schwannoma, intratympanic gentamicin, ménière's disease

## Abstract

Inner ear schwannoma (IES), also known as intralabyrinthine schwannoma, is a rare benign tumor typically presenting with hearing loss, tinnitus, and vestibular symptoms. In some cases, vestibular manifestations mimic Ménière's disease, characterized by recurrent vertigo attacks in which endolymphatic hydrops has been implicated in the underlying pathophysiology. Herein, we report the case of a 44-year-old woman with left-sided IES who developed intractable recurrent vertigo. Despite profound hearing loss in the affected ear, vestibular function testing revealed residual activity in all three semicircular canals on the affected side. Contrast-enhanced magnetic resonance imaging demonstrated a tumor occupying nearly the entire cochlea and partially extending into the vestibule, while no definite endolymphatic hydrops was identified. Medical therapy was ineffective in controlling the vertigo attacks. The patient underwent repeated intratympanic gentamicin (GM) injections. After the fifth session, she experienced transient severe vertigo followed by complete resolution of recurrent vertigo attacks. Follow-up testing demonstrated further reductions in vestibulo-ocular reflex gains in all three semicircular canals on the affected side, while contralateral vestibular function remained preserved. No recurrent vertigo attacks occurred during the two-year follow-up period. This case suggests that intratympanic GM may represent a non-surgical treatment option for selected patients with IES-related refractory vertigo.

## Introduction

Inner ear schwannoma (IES), also referred to as intralabyrinthine schwannoma, is a rare benign tumor originating within the membranous labyrinth. Patients typically present with hearing loss, tinnitus, dizziness, or imbalance. Vestibular symptoms associated with IES are diverse and may include dizziness, positional vertigo, and recurrent vertigo attacks resembling Ménière's disease, which are often refractory to medical therapy. Recent studies using delayed gadolinium-enhanced magnetic resonance imaging (MRI) have reported endolymphatic hydrops in some patients with IES. Endolymphatic hydrops is a pathological enlargement of the endolymphatic space implicated in Ménière's disease and may contribute to recurrent vertigo in patients with IES. Intratympanic gentamicin (GM) reduces peripheral vestibular function through its vestibulotoxic effect and is used to control refractory vertigo in Ménière's disease; however, its role in IES-related vertigo remains poorly established.

Herein, we present a case of IES with intractable recurrent vertigo that was treated with intratympanic GM after the patient declined tumor surgery. Following treatment, additional vestibular hypofunction was objectively demonstrated on the affected side, accompanied by complete resolution of recurrent vertigo attacks. This case illustrates the potential role of intratympanic GM as a non-surgical treatment for selected patients with IES-related refractory vertigo.

## Case presentation

A 44-year-old woman developed left-sided hearing loss 18 years before presentation, which progressed to complete deafness within the following year. Intravenous steroid therapy was administered without improvement, and MRI at that time revealed no abnormalities. One year before presentation, she developed left-sided tinnitus and was evaluated at a local otolaryngology clinic. MRI revealed an IES, and a wait-and-scan approach was adopted. Several months later, she experienced recurrent spontaneous episodes of spinning vertigo lasting several hours and was referred to our hospital for further evaluation.

At her initial visit, pure-tone audiometry revealed normal hearing in the right ear (three-frequency pure-tone average (500, 1,000, and 2,000 Hz), 18.3 dB) and profound hearing loss exceeding 100 dB in the left ear (Figure 1).

**Figure 1:**
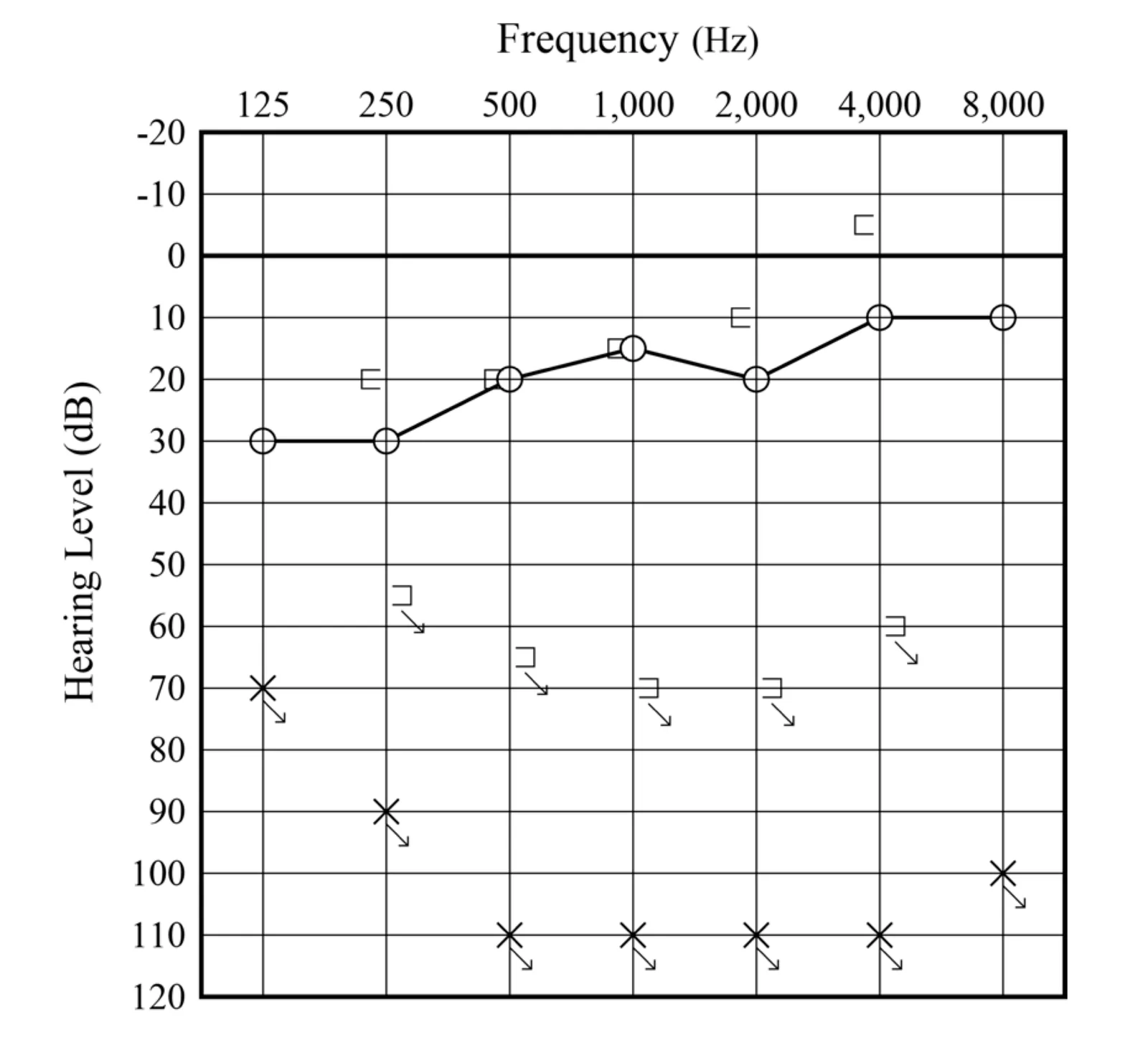
Pure-tone audiogram at the initial visit The right ear showed normal hearing (three-frequency pure-tone average (500, 1,000, and 2,000 Hz), 18.3 dB), whereas the left ear demonstrated profound hearing loss exceeding 100 dB.

No spontaneous nystagmus was observed. Positional testing revealed right-beating and downbeat nystagmus in the supine position and downbeat nystagmus when the head was turned to the left. Neurological examination revealed no focal abnormalities, and brain MRI showed no structural lesions involving the brainstem or cerebellum that could account for the downbeat nystagmus. Caloric testing with cold air (20°C for 60 sec) showed maximum slow-phase velocities of 19°/sec on the right and 10°/sec on the left, corresponding to a canal paresis of 31%. Video head impulse testing (vHIT), performed using the ICS Impulse system (Natus Medical, Taastrup, Denmark), demonstrated reduced vestibulo-ocular reflex (VOR) gain in the left anterior (0.64), lateral (0.59), and posterior (0.52) semicircular canals, with preserved right-sided canal function (Figure 2A). VOR gains below 0.8 and 0.7 were considered abnormal for the lateral and vertical semicircular canals, respectively. Overt corrective saccades were observed in the left lateral semicircular canal.

**Figure 2:**
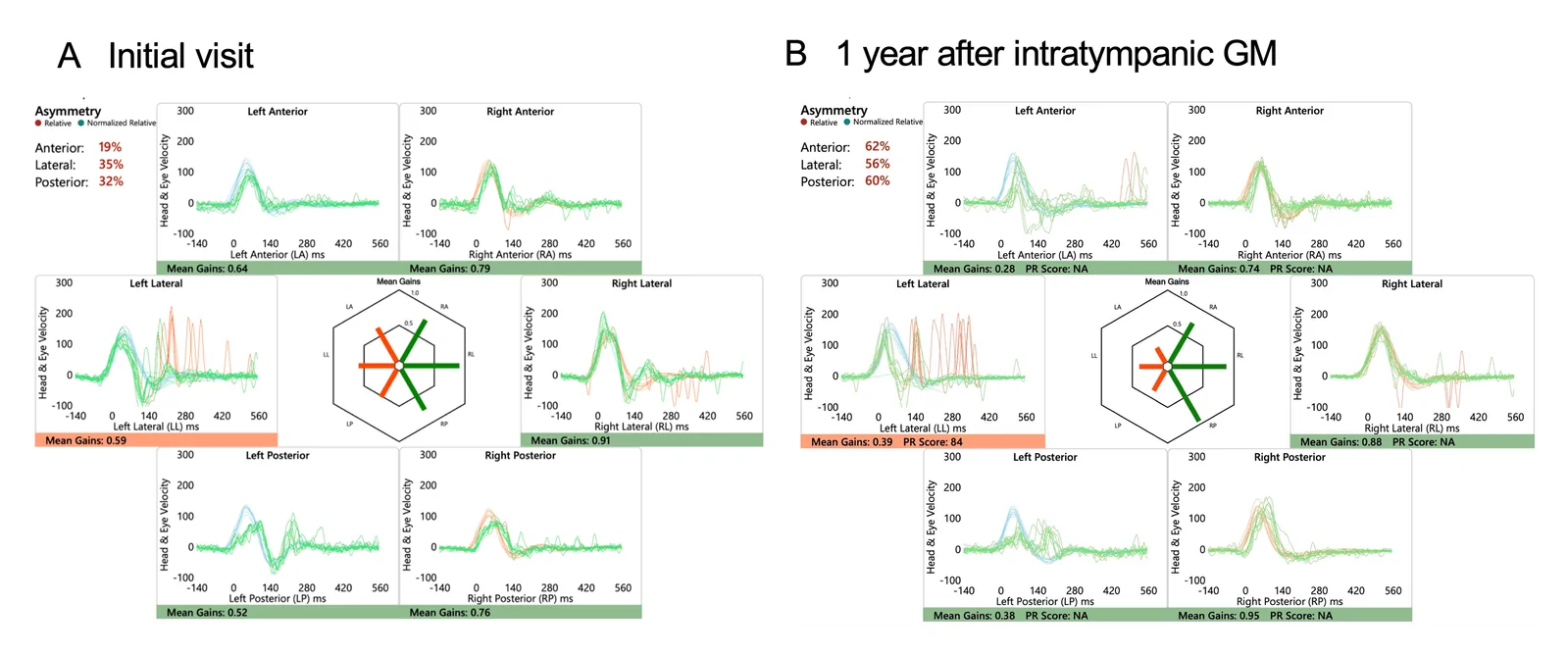
Video head impulse testing (A) At the initial visit, reduced VOR gains were observed in the left anterior (0.64), lateral (0.59), and posterior (0.52) semicircular canals, while VOR gains on the right side were within normal limits. Overt corrective saccades were observed in the left lateral semicircular canal. (B) At the one-year follow-up after intratympanic gentamicin therapy, further reductions in VOR gains were observed in the left anterior (0.28), lateral (0.39), and posterior (0.38) semicircular canals, whereas right-sided canal function remained preserved. Overt corrective saccades were evident in the left lateral semicircular canal. VOR: vestibulo-ocular reflex

Contrast-enhanced T1-weighted MRI revealed an enhancing mass involving nearly the entire left cochlea, extending from the modiolus to the fundus of the internal auditory canal and partially into the vestibule (Figure 3A). MR cisternography demonstrated signal voids in the left cochlea and part of the vestibule, while the semicircular canals remained visible (Figure 3B). HYDROPS imaging, generated by subtraction of a positive endolymph image from a positive perilymph image obtained four hours after intravenous gadolinium administration, did not demonstrate definite endolymphatic hydrops. Evaluation of the endolymphatic space in the affected vestibule was limited by tumor involvement. Two small low-signal areas were observed in the vestibule; however, they could not be confidently interpreted as representing the endolymphatic space because image-subtraction artifacts could not be excluded (Figure 3C).

**Figure 3:**
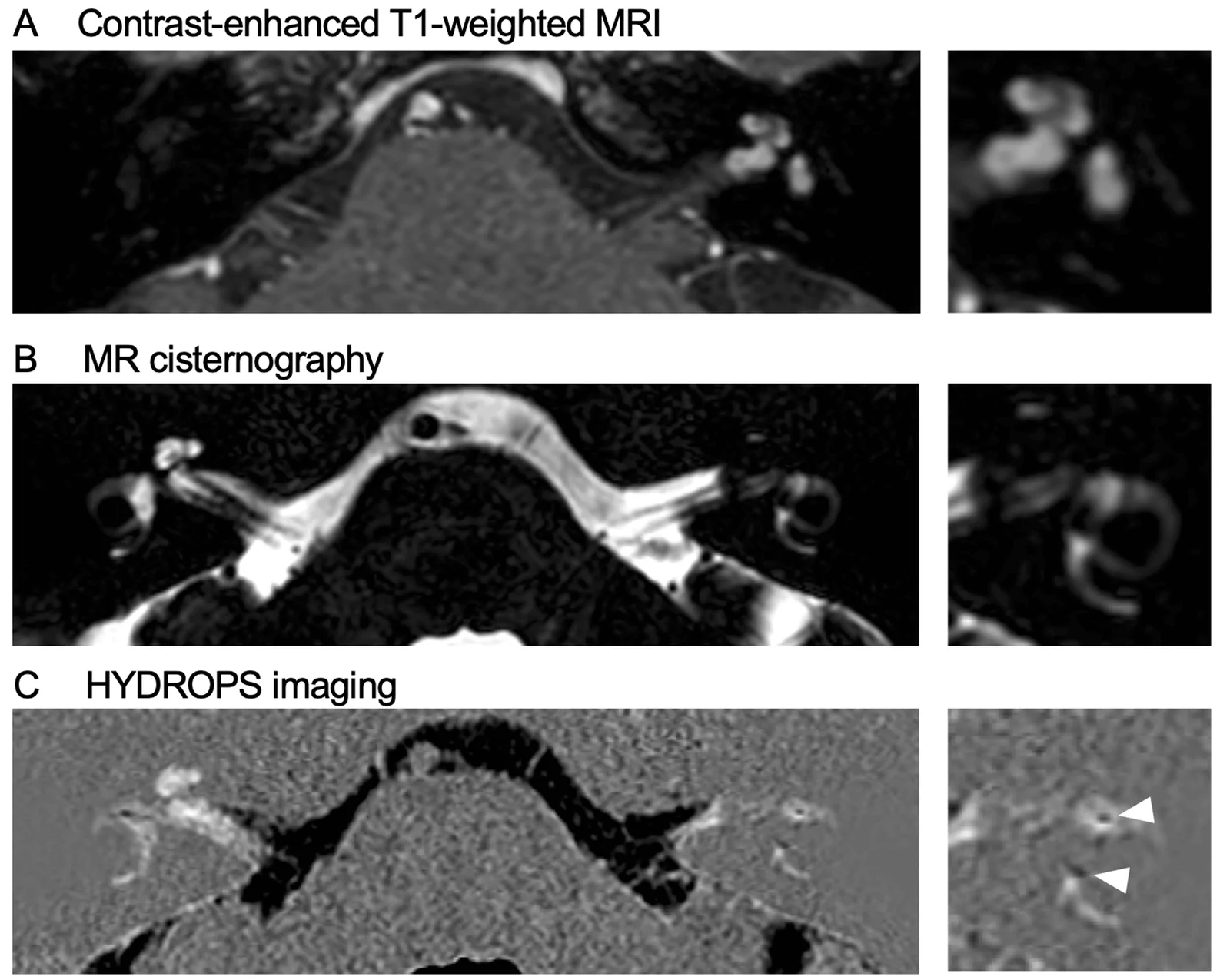
MRI at the initial visit (A) Contrast-enhanced T1-weighted image showing an enhancing mass involving nearly the entire left cochlea, extending from the modiolus to the fundus of the internal auditory canal and partially into the vestibule. (B) MR cisternography demonstrating signal voids in the left cochlea and part of the vestibule, while the semicircular canals remain visible. (C) HYDROPS imaging, generated by subtraction of a positive endolymph image from a positive perilymph image obtained four hours after intravenous gadolinium administration. Definite endolymphatic hydrops was not identified. Two small low-signal areas were observed in the vestibule (arrowheads), but image-subtraction artifacts could not be excluded. The left column displays both ears for comparison; the right column presents magnified views of the left ear with inner ear schwannoma. MRI: magnetic resonance imaging

Based on these findings, differential diagnoses were considered. The vertigo attacks, lasting several hours on average and occurring spontaneously, were not consistent with benign paroxysmal positional vertigo. Vestibular migraine was also considered unlikely because there were no accompanying migraine features. Although definite endolymphatic hydrops was not demonstrated on MRI, medical treatment for vertigo was initiated according to therapeutic strategies for Ménière's disease. Treatment with isosorbide (90 mL/day) and betahistine (36 mg/day) was started and later supplemented with the Kampo medicine ryokeijutsukanto (7.5 g/day); however, the vertigo attacks persisted.

The patient declined surgical tumor removal but desired more effective treatment for the recurrent vertigo. Intratympanic GM therapy was therefore selected as a non-surgical treatment for vertigo. During the six months preceding GM therapy, she experienced seven spontaneous vertigo attacks despite medical treatment (Figure 4). Approximately one year after her first visit, intratympanic GM therapy was initiated. According to our institutional protocol, GM was administered once weekly, with the number of injections determined by the clinical response. The development of marked vertigo after an injection was used as a clinical indicator for treatment cessation. For each treatment, 1 mL of GM solution (60 mg/1.5 mL preparation) was mixed with 0.5 mL of 7% sodium bicarbonate, yielding a final concentration of approximately 26.7 mg/mL. From this mixture, 0.3-0.5 mL was injected into the tympanic cavity. After the fifth injection, the patient experienced marked transient vertigo, which subsequently resolved, and no further injections were administered. Thereafter, no recurrent vertigo attacks occurred during the two-year follow-up period, and the patient remained free of persistent dizziness or unsteadiness (Figure 4).

**Figure 4:**
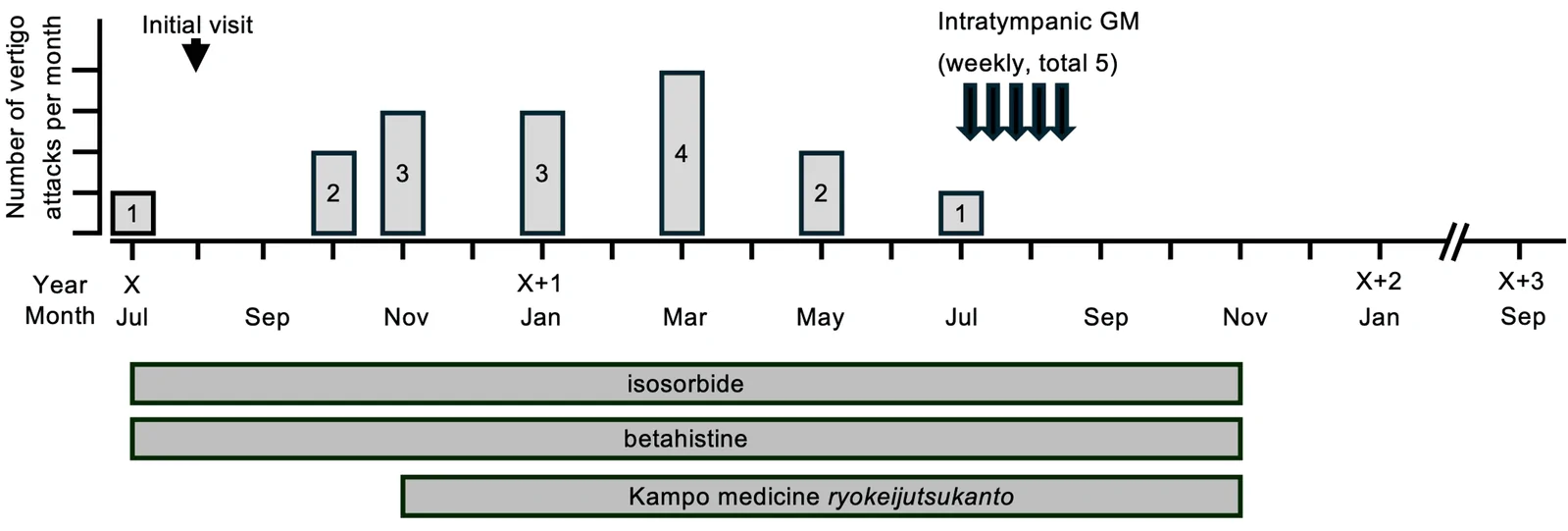
Clinical course and treatment timeline The monthly frequency of vertigo attacks (vertical axis) is plotted against time (horizontal axis). Medical treatment with isosorbide, betahistine, and the Kampo medicine ryokeijutsukanto was initiated but failed to control the vertigo attacks. Intratympanic gentamicin injections (five sessions, indicated by arrows) were administered weekly, after which vertigo attacks resolved. No recurrent vertigo attacks occurred during the two-year follow-up period.

At the one-year follow-up, vHIT showed further reductions in left canal gains (anterior 0.28, lateral 0.39, posterior 0.38), while right-sided canal function remained intact (Figure 2B).

## Discussion

The incidence of IES is estimated at 1.1 per 100,000 population, increasing with age to 4.1 per 100,000 in individuals over 70 years [1]. At diagnosis, over 90% of patients present with hearing loss, 69.1% experience tinnitus, and 59.1% report vestibular symptoms [2]. Vestibular symptoms in IES include a variety of manifestations, such as dizziness, positional vertigo, and Ménière's disease-like vertigo attacks [2], which are often intractable [3].

Several MRI-based reports have documented cases of IES complicated by endolymphatic hydrops [4-7]. These cases typically presented with recurrent Ménière's disease-like vertigo [4]. Hydrops formation was observed in both the cochlea and vestibule [4-6], and treatment options included either observation or surgery [4,5]. In surgically treated cases, no recurrence of vertigo was observed during follow-up [4-6], whereas in conservatively managed cases, vertigo did not worsen [4]. The formation of hydrops in IES is hypothesized to result from tumor-induced obstruction of endolymphatic flow [5].

In the present case, the patient presented with unilateral profound sensorineural hearing loss accompanied by recurrent vertigo. Contrast-enhanced MRI revealed an IES involving nearly the entire cochlea and partially extending into the vestibule, without involvement of the semicircular canals. Definite endolymphatic hydrops was not identified on HYDROPS imaging. However, evaluation of the vestibular endolymphatic space was limited by partial tumor involvement. In addition, two small low-signal areas were observed in the vestibule, but these could not be confidently interpreted as representing the endolymphatic space because image-subtraction artifacts could not be excluded. Therefore, subtle endolymphatic hydrops cannot be completely excluded in this case. Given previous reports of endolymphatic hydrops associated with IES [4-7], tumor-related disturbance of endolymphatic flow remains a possible mechanism underlying the recurrent Ménière's disease-like vertigo in this patient.

Intratympanic GM injection suppresses vertigo attacks by reducing peripheral vestibular function through its vestibulotoxic effect [8]. However, cochlear hair cells may also be affected, posing a risk of worsening hearing loss [8]. Treatment options for IES-related vertigo include surgery and intratympanic GM injection [4,9]. Notably, one case of IES with recurrent vertigo and fluctuating hearing loss achieved symptom relief following a single intratympanic GM injection, with vHIT confirming reduced vestibular function [9]. Prophylactic intratympanic GM injection prior to tumor resection has also been reported to suppress postoperative vertigo symptoms [10].

A notable finding in the present case was the objective change in vestibular function after intratympanic GM therapy. Before treatment, residual VOR responses were present in all three semicircular canals on the affected side despite profound hearing loss. At the one-year follow-up, VOR gains had further decreased in all three affected semicircular canals, whereas contralateral vestibular function remained preserved. This additional ipsilesional vestibular hypofunction was accompanied by complete resolution of recurrent vertigo attacks, with no recurrence during the two-year follow-up period. Although a causal relationship cannot be established from a single case, these findings suggest that residual peripheral vestibular activity in the affected ear may have contributed to the recurrent vertigo attacks.

In recent years, cochlear implantation has emerged as an effective intervention for single-sided deafness. In IES, cochlear implantation is considered a feasible option regardless of whether the tumor is resected [11,12]. When the cochlear nerve is preserved, auditory rehabilitation through cochlear implantation represents a promising option [13]. From an auditory rehabilitation perspective, IES has recently been categorized into six subtypes based on tumor location: intravestibular, intracochlear, intravestibulocochlear, transfundal without modiolar involvement, transfundal with modiolar involvement, and multifocal [14]. The present case corresponds to the transfundal with modiolar involvement subtype. By avoiding surgical tumor resection, cochlear implantation remains a potential option for future auditory rehabilitation in this patient. However, cochlear nerve function was not directly assessed, and favorable auditory outcomes cannot be assumed given the long duration of deafness, extensive cochlear and modiolar involvement, and extension to the fundus of the internal auditory canal.

This report has several limitations. First, the diagnosis of IES was based on characteristic MRI findings without histopathological confirmation because tumor resection was not performed. Second, subtle endolymphatic hydrops could not be completely excluded because evaluation of the vestibular endolymphatic space was limited by tumor involvement and potential image-subtraction artifacts. Third, this is a single case, and the temporal association between additional ipsilesional vestibular hypofunction and resolution of vertigo attacks does not establish causality. Finally, cochlear nerve function and the potential outcome of future cochlear implantation were not directly assessed.

## Conclusions

This case represents a rare presentation of IES with intractable recurrent vertigo treated with intratympanic GM injection. Following treatment, additional vestibular hypofunction was objectively demonstrated in all three semicircular canals on the affected side, accompanied by complete resolution of recurrent vertigo attacks. Intratympanic GM may serve as a non-surgical treatment option for selected patients with IES-associated intractable vertigo refractory to conventional medical treatment.
